# Study on the Wear Performance of Brake Materials for High-Speed Railway with Intermittent Braking under Low-Temperature Environment Conditions

**DOI:** 10.3390/ma15248763

**Published:** 2022-12-08

**Authors:** Lei Ma, Siyuan Ding, Chao Zhang, Meixian Zhang, Hanbo Shi

**Affiliations:** 1 School of Mechanical Engineering, Key Laboratory of Fluid and Power Machinery, Ministry of Education, Xihua University, Chengdu 610039, China; 2 School of Mechanical Engineering, Southwest Jiaotong University, Chengdu 610031, China

**Keywords:** low-temperature environment, intermittent braking, high-speed railway brake materials, wear

## Abstract

The pin on the disc friction tester was used to conduct the intermittent braking testing of train brake materials with a low-temperature environment simulation device at temperatures 20 °C, 0 °C, −10 °C, −20 °C, and −30 °C. The results show that intermittent braking presents different wear characteristics of braking materials at low temperatures. Under different ambient temperature conditions, the most volatile friction coefficient caused by intermittent braking happens at 0 °C, and the wear rate of brake materials reaches its maximum at 0 °C. The wear surface morphology of the brake pad material mainly includes scratches, furrows, adhesions, and abscission pits, while the surface of the brake disc material was dominated by scratches, furrows, and adhesions. With the decrease in temperature, the adhesion damage of the brake pad/disc material increases. At 0 °C, the brake pad material has crack damage.

## 1. Introduction

Because railway transportation is characterized by high speeds, low energy consumption, a large capacity, and a high degree of safety, it has become an important mode of transportation worldwide and occupies a very important position in the national economic development of each country [1,2]. Disc braking, which consumes the kinetic energy of a high-speed train via disc–pad friction, is one of the important approaches to train deceleration and braking, as well as safety and performance assurance [3,4]. The brake disc is the most critical component of the brake system, so the safety assessment and fault prevention of the high-speed train brake mechanism are required to be higher [5,6]. However, with the rapid development of high-speed trains and the diversification and complexity of the environment, some new operational problems have gradually emerged in front of scientists. Among them, natural environmental factors are typical examples, such as high temperatures, very cold temperatures, high humidity, and low humidity. In particular, the problem of high-speed trains running in very cold areas is particularly prominent [7,8]. The temperature of some alpine areas of the railway service can be as low as −30 °C in the winter. On the one hand, the braking performance of the high-speed railway is related to the braking material; on the other hand, the braking form, such as continuous braking or intermittent braking, can also affect braking performance [9]. As the core components of the railway braking system, the brake disc and the brake pad are the keys to ensuring the safe operation of the railway [10]. In the process of high-speed train braking, any abnormal friction conditions will produce great potential safety hazards, which will endanger driving safety. During braking, the mechanical energy of the high-speed train will be converted into heat energy in a very short time, and a small part of this heat energy will volatilize into the air, and most of the heat energy will be absorbed by the brake disc [11]. According to data, during the whole braking process, the surface temperature of the brake disc can rise to 700 °C [12,13,14]. In the process of cooling, the disc surface temperature will form a typical step cooling, and a thermal stress difference will be formed between the two steps. If the cycle continues for a long time, thermal cracks will be formed at the two steps, which will reduce braking performance [15,16].

The influence of the environment on the braking performance of the high-speed railway cannot be ignored. Many researchers have studied brake disc wear under extreme environments [17]. For example, Kang jie Rong et al. studied the wear of wheel/rail brake materials under high temperatures and humid environments. The research results show that humidity has a significant impact on the wear behavior of brake materials. Under high temperature and low humidity environments, fatigue wear is the main wear mode. With the increase in humidity, abrasive wear becomes more and more serious and gradually becomes the main wear [18]. Qian Kuncai et al. simulated the braking process of a high-speed train braking system under rain and snow conditions by building a 1:1 friction and wear braking test bench and studied the friction and wear performance of braking materials and the reasons for the decline of braking performance. The test results showed that the friction coefficient between the brake material surfaces increases with the increase in brake speed and pressure in rain and snow [19]. Eriksson, a foreign scholar, and others studied the friction mechanics test with ambient air humidity as a variable. It was shown that air humidity has an obvious impact on the friction coefficient and wear loss of friction materials, and the impact is extremely critical. When the humidity in the air is 20~80%, the influence of humidity on the friction coefficient has a certain limit, but when the humidity is greater than 80%, the friction coefficient will increase with the increase in humidity [20].

At the same time, many scholars use finite element software and numerical simulation methods to study and analyze environmental factors. For example, scholars Xing Wu and others built a model to analyze the severe wear process of the brake disc in an icing environment, which saved considerable time and costs [21]. Ulf Olofsson et al. have developed a laboratory simulation test method, which is mainly used to evaluate and test the braking capacity of brake disc materials of high-speed trains under a winter snow environment [22]. Lyu Yezhe studied the friction and wear properties of three types of brake materials under low-temperature conditions using a pin on disc friction and wear tester and a low-temperature simulator. The results showed that the composite material had the best friction performance [23].

To summarize, the researchers’ research focus and novelty is the research on the influence of the third medium on the braking performance of high-speed train braking materials in the winter, while few people study the friction and wear performance of intermittent braking materials from the ambient temperature itself.

In this study, we conducted a brake simulation test in a low-temperature environment using a pin on the disc friction test machine with a low-temperature control device. We observed the wear rate, average friction coefficient, and surface morphology of the brake disc/pad material specimens. The influence of low temperature on friction and wear properties of brake disc materials under intermittent braking conditions were analyzed. The research results provide a theoretical basis for the safety braking of a high-speed railway in the alpine region

## 2. Experimental Details

### 2.1. The Materials

**Figure 1:**
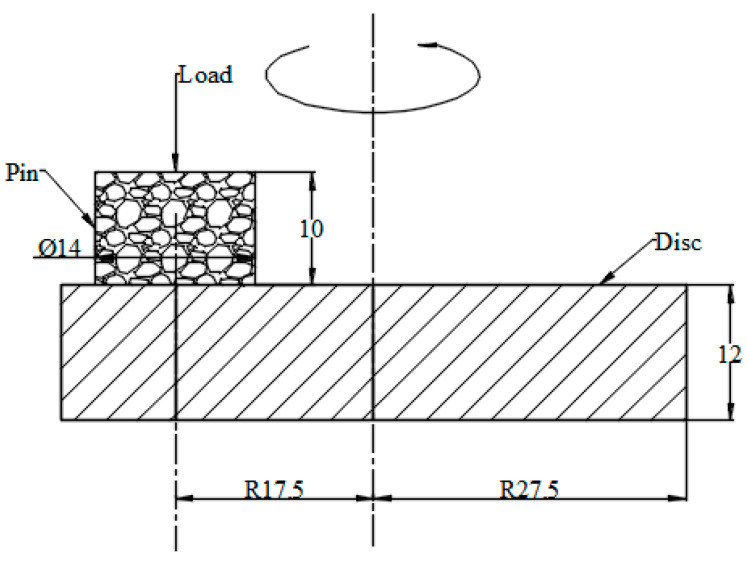
The size of the disc and pin samples.

**Figure 2:**
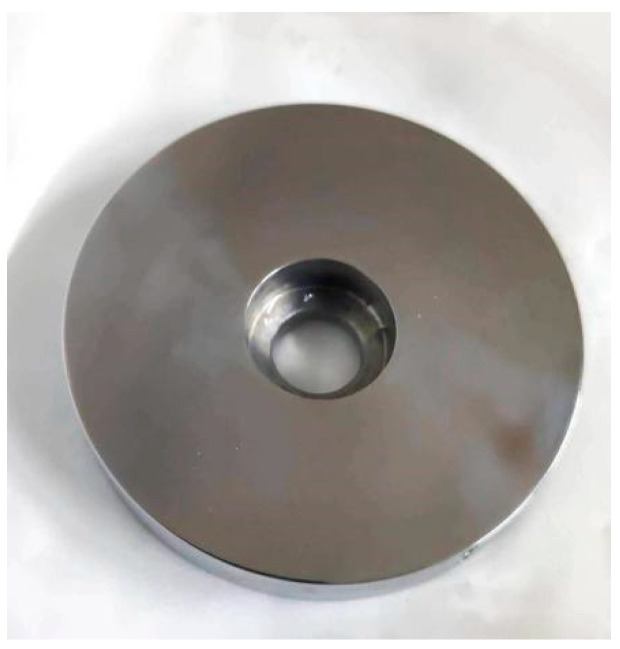
Brake disc sample.

The materials are all brake materials cut on-site for high-speed trains (CRH380, 350 km/h) in China. The main component of the brake disc material is forged steel, and the main component of the brake pad material is powder metallurgy [24]. Table 1 shows the elemental composition of the brake disc and brake pad materials. Figure 1 exhibits the size of the disc and pin samples. The pin specimen was made from a copper-based powder alloy used by a brake pad material in the field, and the disc specimen was made of a forged steel brake disc material used in the field, as shown in Figure 2. The initial surface roughness (Ra) of the brake disc was 1.00 ± 0.20 μm. The initial surface roughness (Ra) of the brake pad was 0.6 ± 0.02 μm.

### 2.2. The Test Equipment

**Figure 3:**
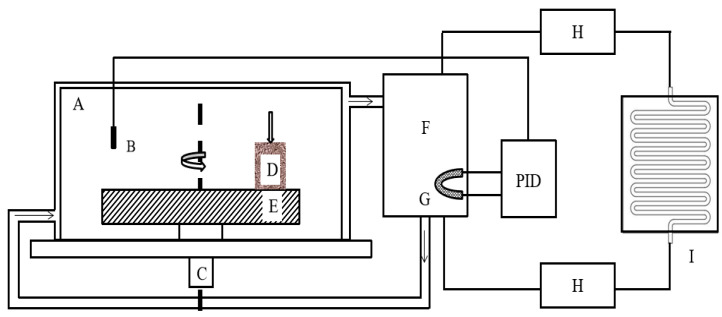
Schematic diagram of the experimental setup: (A) temperature-controlled climate chamber; (B) sensor; (C) base; (D) pin specimen; (E) disc specimen; (F) alcohol tank; (G) red heating plate; (H) transmission line; (I) condenser.

After completing the steps of sample preparation, cleaning, drying and weighing, the calibration and calibration of the equipment were performed. Finally, installing the sample on the test equipment was set in advance. The testing machine and cryogenic device were turned on and waited until the ambient temperature was stable before proceeding to start working. Figure 3 is a simple schematic diagram of the test equipment and cryogenic device. The pin specimen (D) contacts the disc specimen (E) with an adjustable rotational speed by a vertically applied brake pressure. The climate chamber (A) is a thermal insulation material that is used for thermal insulation and prevents the entry of the third medium while ensuring a continuous and stable temperature. The main function of the alcohol tank (F) and condenser (I) is to provide a cooling medium. The sensor (B) monitors the temperature change in the climate chamber in real time so as to feedback to the control system in time. Through the cooperation of the above steps, a stable ambient temperature can be obtained in the cavity.

### 2.3. Test Process

The friction wear test of intermittent braking was carried out at a low temperature, and the test parameters were set, as shown in Table 2. By comparing the experimental data with the field data, it was found that a low load and a low sliding speed will not cause a change in the friction coefficient and wear rate of the pin and disc. [25,26,27]. Taking the initial braking speed of a high-speed train as 250 km/h and the braking distance as 3000 m, as an example, in the same braking time, the linear speed of the pin in the pin disc test is 0.5 m/s, and the work consumed per unit area of the two is the same [7]. According to the calculation and references, the sliding speed is 0.5 m/s, and the contact pressure is 1.0 MPa. Before the test, the samples were polished with 180 mesh, 600 mesh, and 1000 mesh sandpapers in order to obtain a surface with a roughness of about 0.1 μm, and then cleaned in an ultrasonic cleaner, and finally dried in a drying oven for 8 h. After 20 min of initial wear on the specimen, then the contact mode for the pair of frictional sub-contract for 10 min, and then separated idle for 10 min, and finally 12 sliding contacts, the total wear accumulated time of 120 min for each group was repeated 3 times. Before the test, the ultrasonic cleaner and alcohol were used to clean the test sample for 15 min. The purpose is to clear the third medium on the brake’s surface. After three times of cleaning, the blower and drying cabinet was used to dry for 6 h to minimize the error caused by weighing. An electronic balance was used with an accuracy of 0.0001 g to measure the mass before and after the test. When observing the surface damage morphology after the test, the following equipment was used: an optical microscope (Olympus bx60 m) made in Japan and a scanning electron microscope (SEM) (phenom pro) made in the Netherlands. The above tests were repeated three times.

### 2.4. Test Method

Define the wear rate α for one revolution of the brake disc specimen as shown in Equation (1):

(1) $$\alpha =\frac{\Delta m}{n}$$

where α is the specimen wear rate (μg/r); Δm is the change in mass of the specimen (μg); and n is the number of rotation cycles of the disc sample (r).

## 3. Results

### 3.1. Friction Coefficient

**Figure 4:**
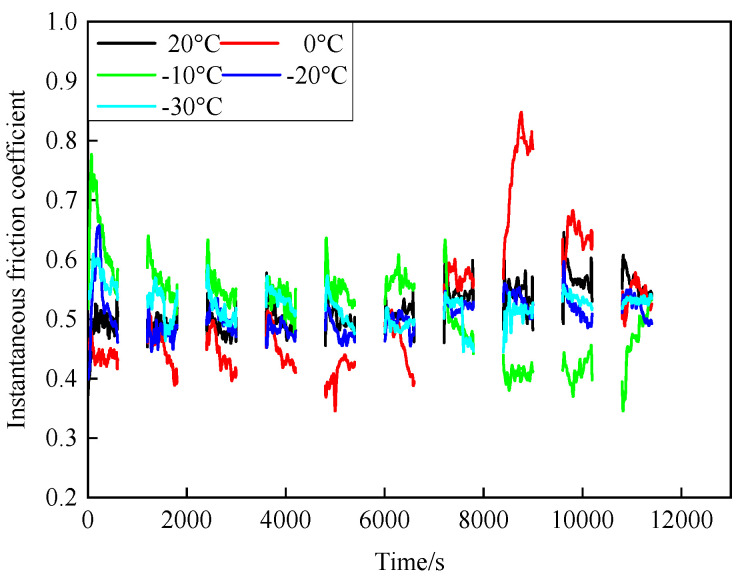
Instantaneous friction coefficient curve.

**Figure 5:**
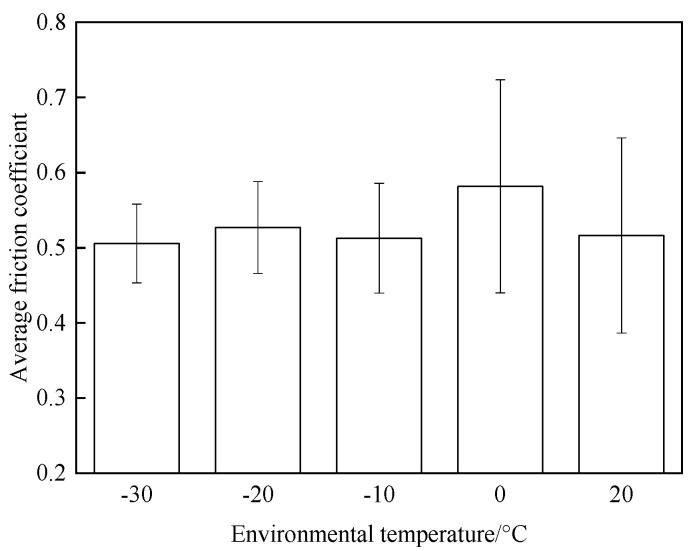
Average friction coefficient histogram.

Figure 4 and Figure 5 show the change in the friction coefficient at low temperatures. It can be seen from Figure 4 that under various temperature conditions, the instantaneous friction coefficient of intermittent braking generally fluctuated greatly, but during 10 min of operation, the instantaneous friction coefficient under various temperatures showed a trend of first increasing and then decreasing. Between 0 s and 7000 s, the instantaneous friction coefficient at −10 °C was larger than at other temperatures, while the instantaneous friction coefficient at 0 °C was the smallest. However, after 7000 s, the instantaneous friction coefficient of the two temperatures showed opposite changes, and the instantaneous friction coefficient reached the maximum at 0 °C (0.85).

In order to further investigate the effect of intermittent braking on the friction coefficient, the results of three repeated tests were averaged, and the results are shown in Figure 5. During intermittent braking, the average friction coefficient at 0 °C reached the maximum value, about 0.585, while the average friction coefficient at other temperatures had little difference. The average friction coefficients at 20 °C, −10 °C, −20 °C, and −30 °C are 0.516, 0.513, 0.527, and 0.506, respectively, which are 11.8%, 12.3%, 9.9%, and 13.5% lower than the average friction coefficients at 0 °C.

### 3.2. Wear Rate

**Figure 6:**
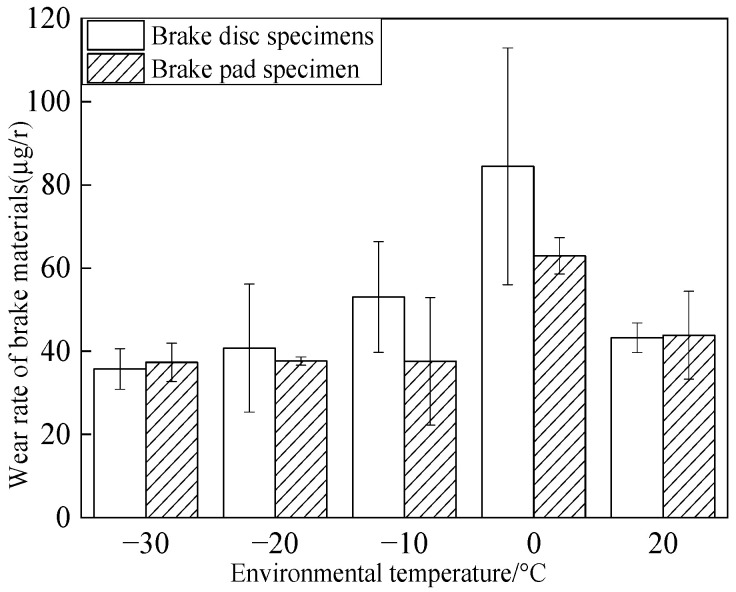
Wear rate of brake materials at different temperatures.

Figure 6 shows the influence of intermittent braking on the wear rate of the brake disc/pad material under a low-temperature environment. The decrease in ambient temperature first caused an increasing and then a decreasing trend of wear rate of the brake disc and reached the maximum value (84.5 μg/r) at 0 °C. Under the ambient temperatures of 20 °C, −10 °C, −20 °C, and −30 °C, the wear rates of brake disc materials were 44.5 μg/r, 54.4 μg/r, 41.3 μg/r, and 36.5 μg/r, respectively, which were 47.3%, 35.5%, 51.1%, and 56.7% of the wear rates of brake disc materials under the ambient temperature of 0 °C, respectively. The changing trend of the brake pad material wear rate was different from that of the brake disc material wear rate. With the temperature dropping from 20 °C to −10 °C, the wear rate of the brake pad material first increased and then decreased, while when the temperature dropped from −10 °C to −30 °C, the wear rate of the brake pad material gradually stabilized. The wear rate of the brake materials was at the maximum at 0 °C with 45.5 μg/r at 20 °C for the brake pad. While at the ambient temperature of −10 °C~ −30 °C, the wear rate of the brake pad material was about 36.9 μg/r.

### 3.3. Surface Morphological Damage

**Figure 7:**
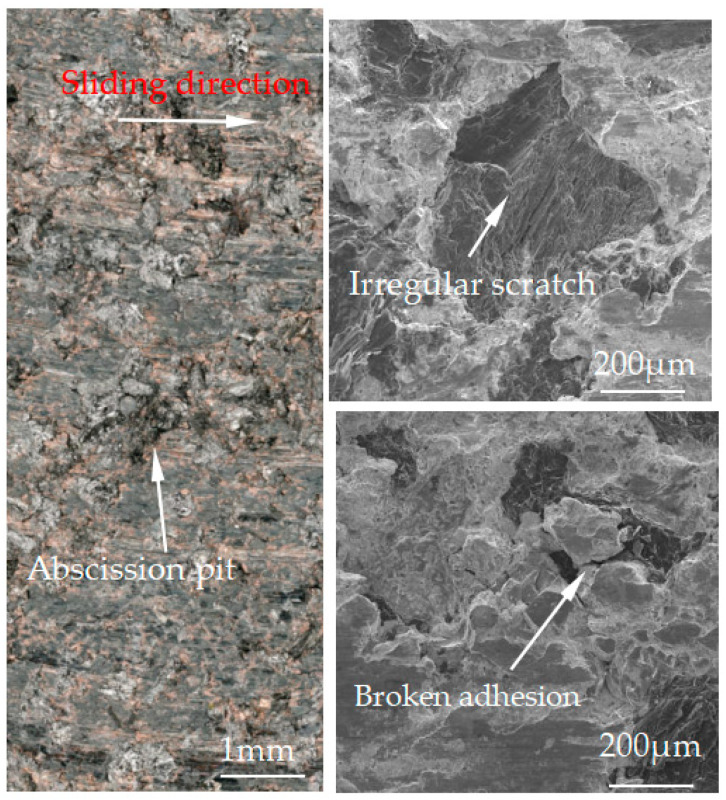
Surface morphology of the pad material after intermittent braking at an ambient temperature of 20 °C.

**Figure 8:**
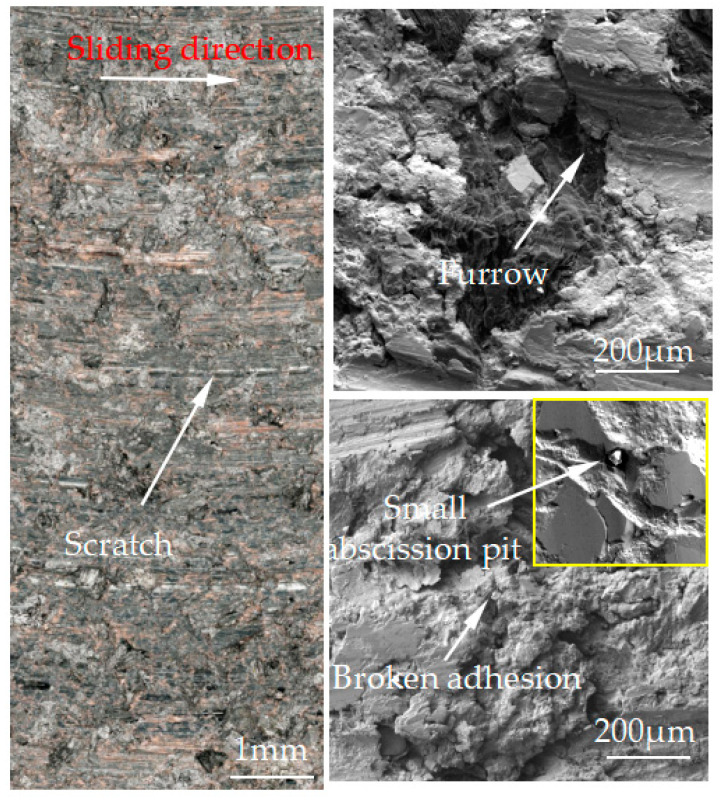
Surface morphology of the pad material after intermittent braking at an ambient temperature of 0 °C.

**Figure 9:**
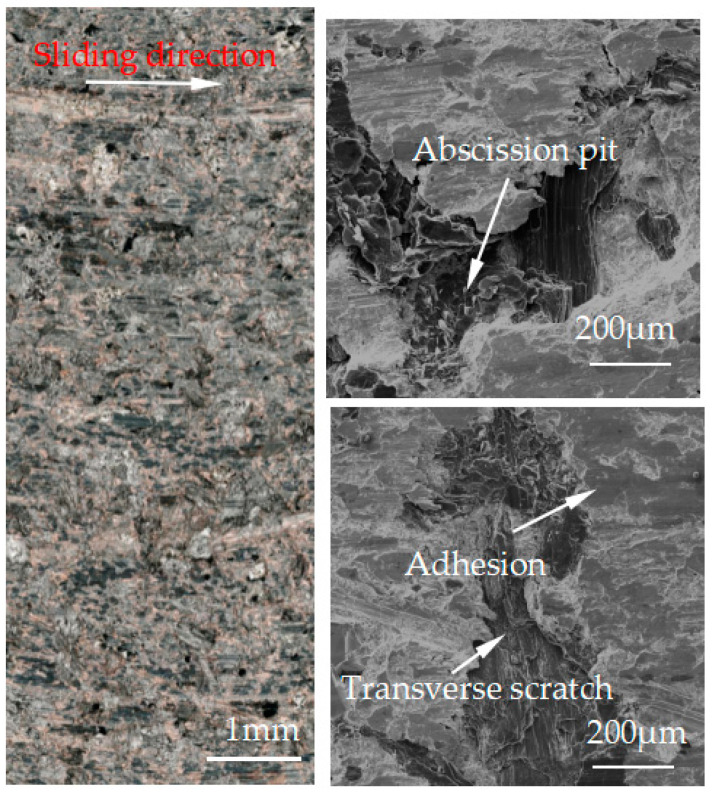
Surface morphology of the pad material after intermittent braking at an ambient temperature of −10 °C.

**Figure 10:**
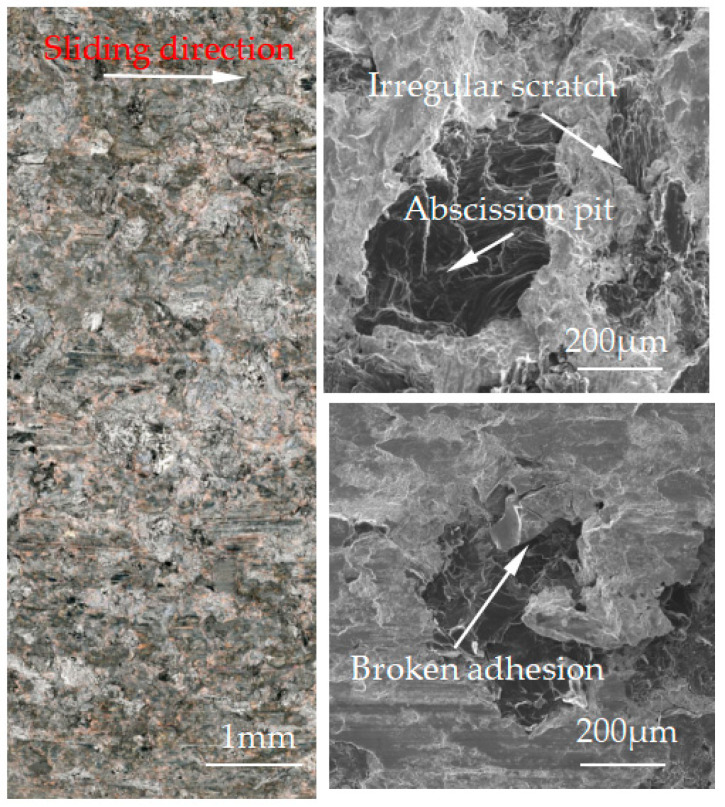
Surface morphology of the pad material after intermittent braking at an ambient temperature of −20 °C.

**Figure 11:**
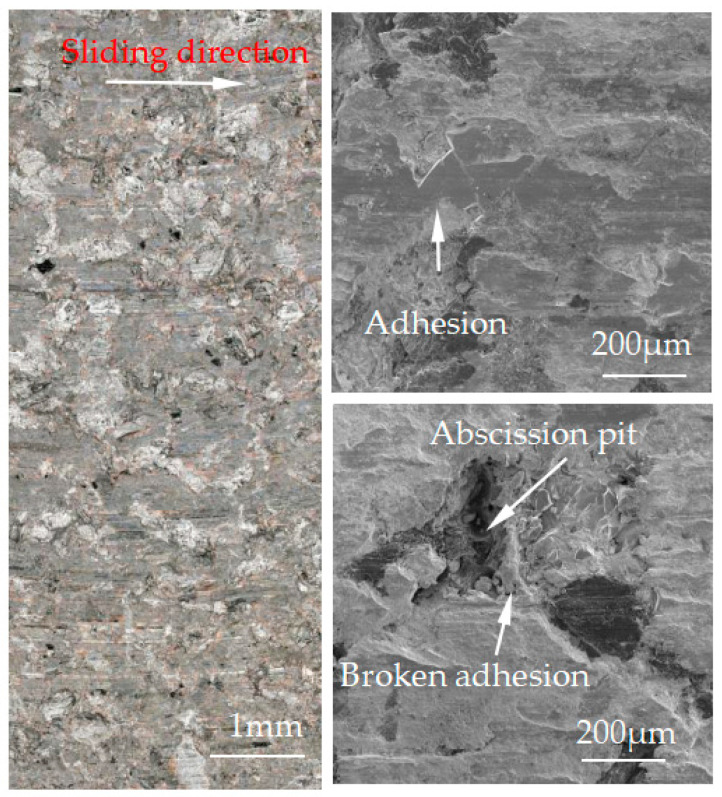
Surface morphology of the pad material after intermittent braking at an ambient temperature of −30 °C.

Figure 7, Figure 8, Figure 9, Figure 10 and Figure 11 show the surface damage morphology of the brake pad material during intermittent braking under different temperature conditions. At various ambient temperatures, the forms of damage on brake pad materials mainly included furrows, irregular scratches, abscission pits, broken adhesions, and other damages. At the ambient temperatures of 20 °C (Figure 7), −20 °C (Figure 10), and −30 °C (Figure 11), the surface of the brake pad material sample was mainly composed of small abscission pits, small scratches and adhesions. At 0 °C (Figure 8), the area and number of abscission pits on the surface of the brake pad material sample increased significantly, and there were obvious irregular scratches and furrows. At −10 °C (Figure 9), the number of adhesions on the surface of the brake pad material sample rose, and the abscission pits and scratches were reduced.

**Figure 12:**
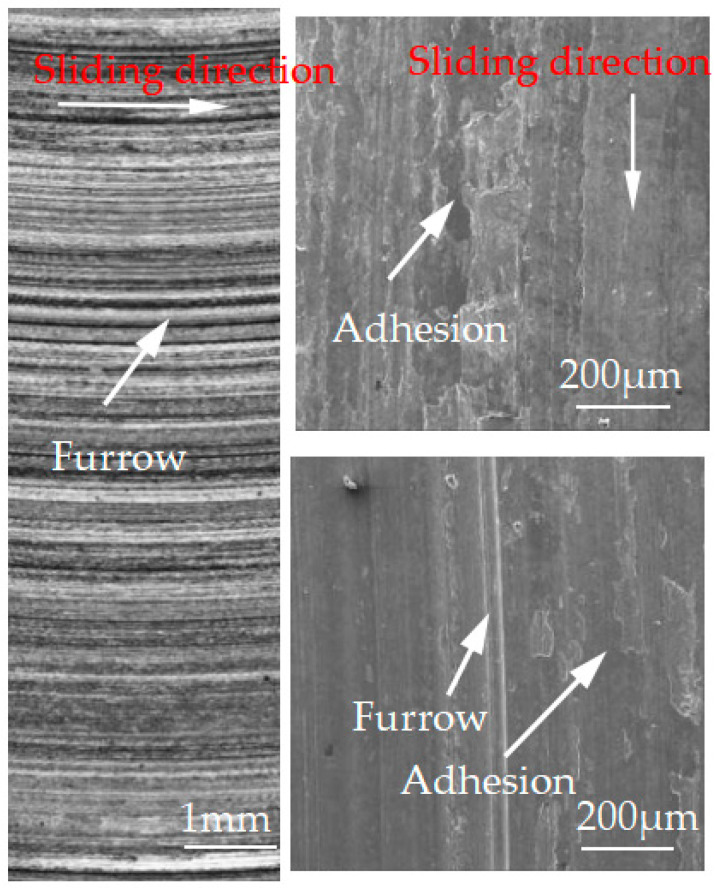
Surface morphology of the disc material after intermittent braking at an ambient temperature of 20 °C.

**Figure 13:**
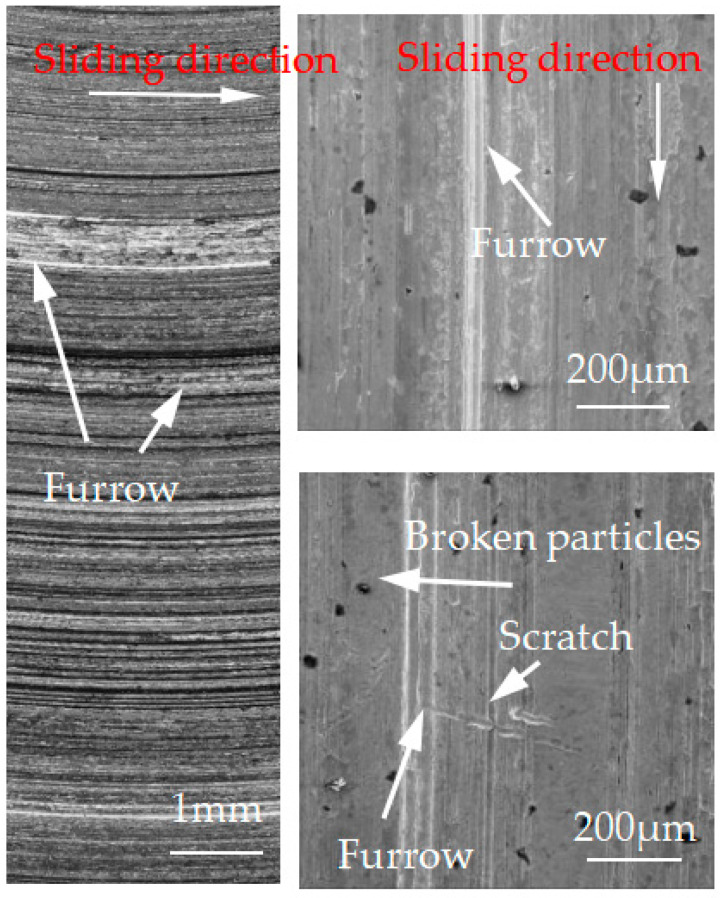
Surface morphology of the disc material after intermittent braking at an ambient temperature of 0 °C.

**Figure 14:**
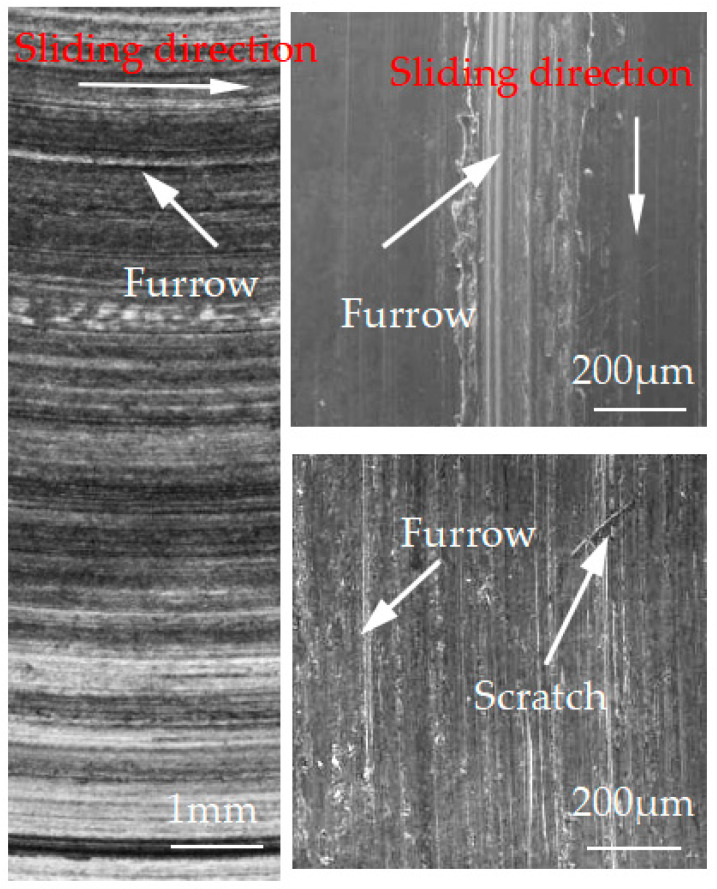
Surface morphology of the disc material after intermittent braking at an ambient temperature of −10 °C.

**Figure 15:**
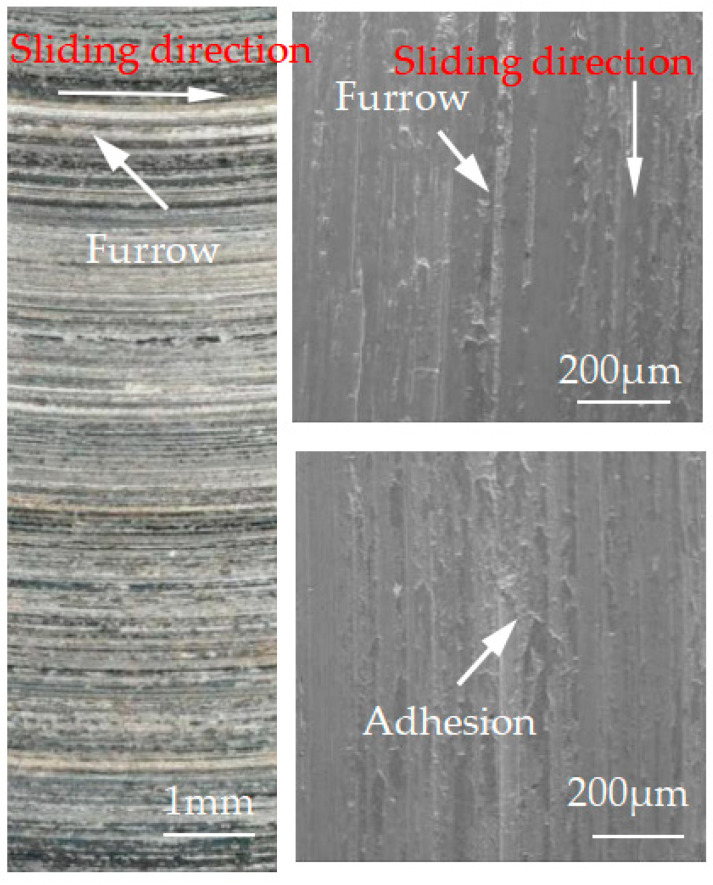
Surface morphology of the disc material after intermittent braking at an ambient temperature of −20 °C.

**Figure 16:**
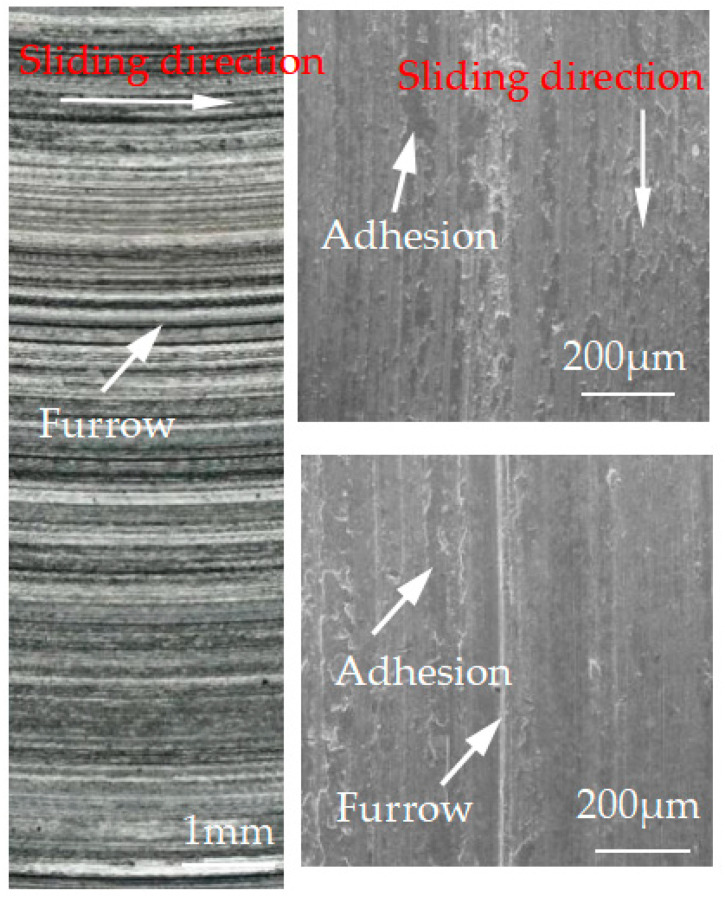
Surface morphology of the disc material after intermittent braking at an ambient temperature of −30 °C.

Figure 12, Figure 13, Figure 14, Figure 15 and Figure 16 show the surface damage morphology of the brake disc material during intermittent braking under different temperature conditions. At various ambient temperatures, the forms of damage on brake disc materials mainly included furrows, scratches, and adhesions. At 20 °C (Figure 12), the surface of the brake disc sample had furrows and a large amount of adhesive. At 0 °C (Figure 13), there were many broken particles, deep furrows, and scratches on the surface of the brake disc sample, and the broken particles were the main reason for the large number of furrows. No obvious adhesive was found on the surface of the sample. At the ambient temperatures of –10 (Figure 14), −20 (Figure 15), and −30 °C (Figure 16), there were still obvious furrows on the surface of the brake disc sample; at the same time, there were also obvious adhesives on the surface.

**Figure 17:**
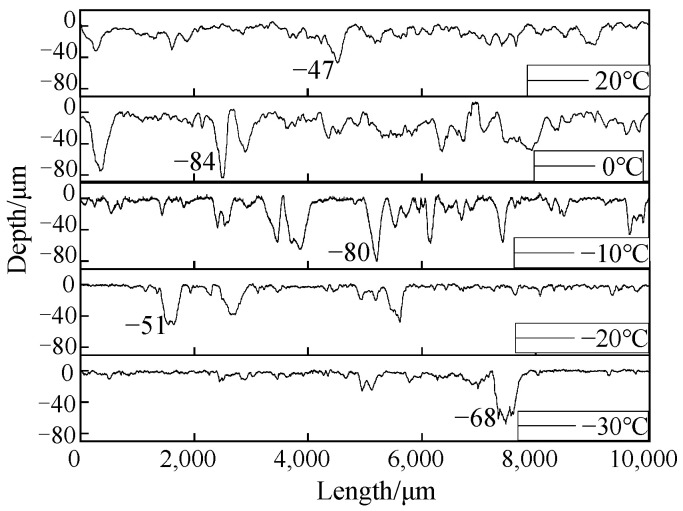
Surface profile of the brake disc braking material.

**Figure 18:**
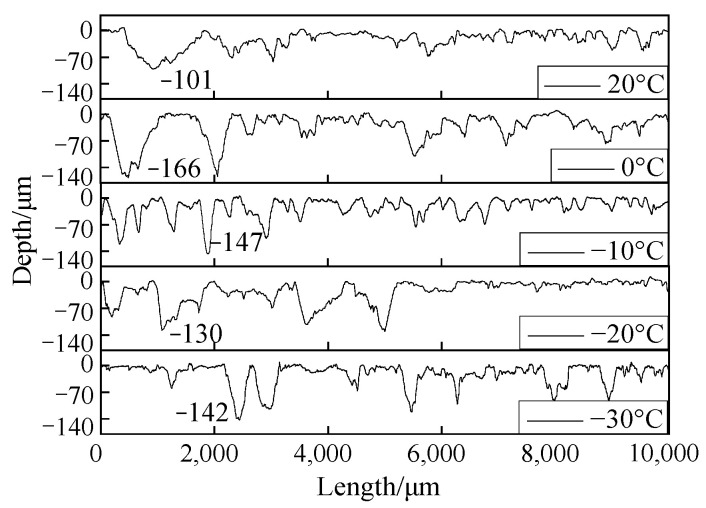
Surface profile of the brake pad braking material.

A surface profiler (JB-6C, China) was used to measure the worn surface profile of the brake disc and brake pad samples perpendicular to the direction of the wear scar. The measurement length was 10 μm. The drawing results are shown in Figure 17 and Figure 18. It can be seen from Figure 17 that the surface profile at 20 °C was relatively flat compared to other temperatures. However, at 0 °C and −10 °C, a large area of furrows and scratches appeared on the surface profile, and the profile depth was significantly deepened. The maximum depth at 0 °C and −10 °C was about 84 μm and 80 μm, respectively. With the decrease in temperature, the depth of the surface profile decreased at −20 °C and −30 °C, and large furrows appeared elsewhere. It can be seen from Figure 18 that the overall damage of the brake lining was relatively obvious compared with that of the brake disc. The brake pad was relatively flat at 20 °C, and the maximum wear scar depth was 101 μm. The wear scar depth of the brake pad was the most serious at 0 °C, and the maximum depth was 166 μm. Under the environment of −10 °C~−30 °C, the wear scar depth of the brake pad decreased in turn.

### 3.4. Subsurface Damage

**Figure 19:**
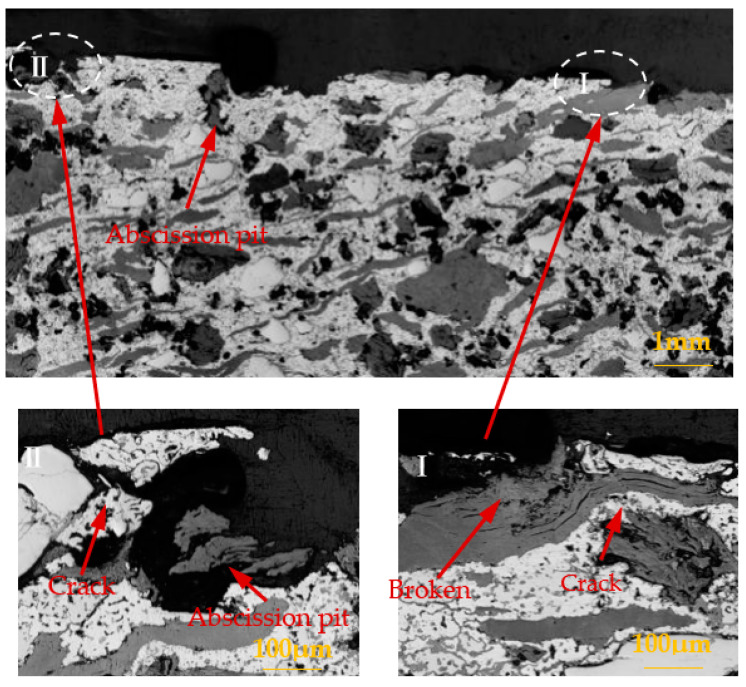
Profile photos of the brake pad material after intermittent brake wear at 0 °C.

**Figure 20:**
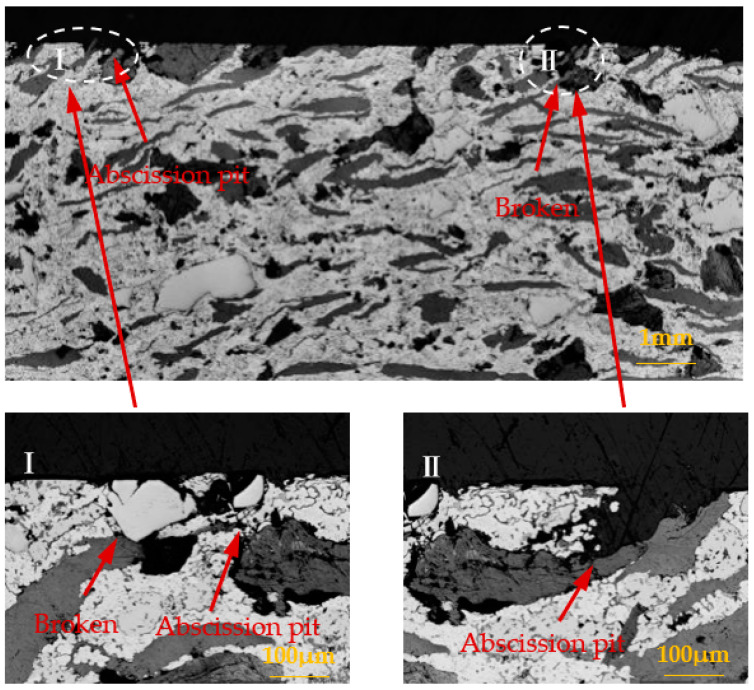
Profile photos of the brake pad material after intermittent brake wear at −30 °C.

**Figure 21:**
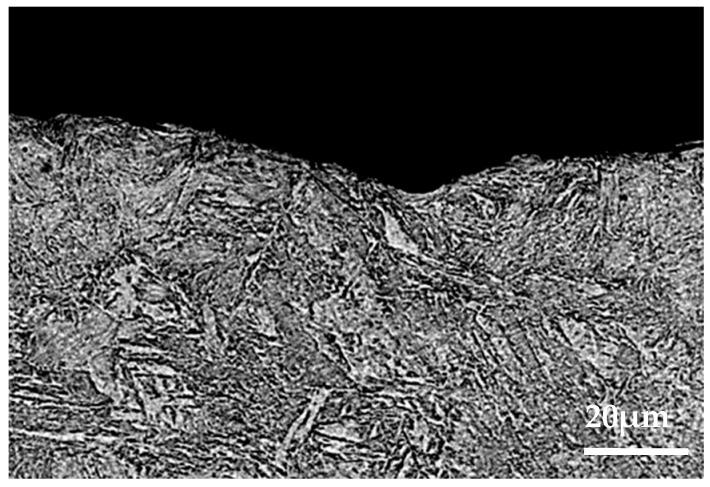
Profile photo of the disc material after intermittent brake wear at an ambient temperature of 0 °C.

**Figure 22:**
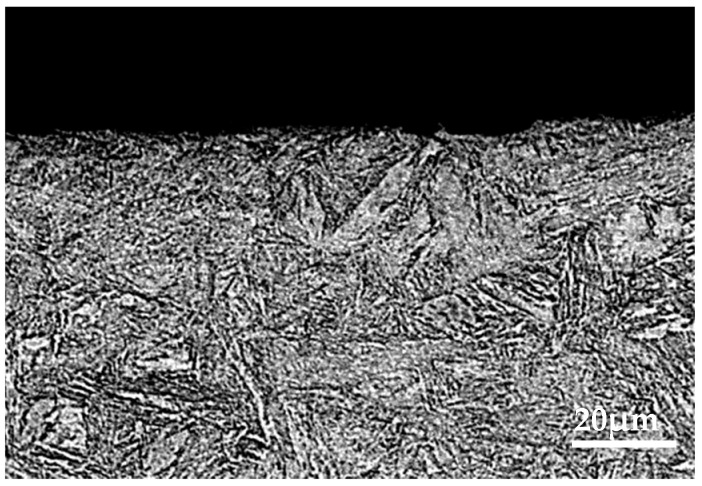
Profile photo of the disc material after intermittent brake wear at an ambient temperature of −30 °C.

Through the analysis of Section 3.1, Section 3.2, and Section 3.3, it was found that the brake material was most seriously damaged under the environment of 0 °C. In order to further analyze the damage mechanism of intermittent braking on brake pad and brake disc materials under a low-temperature environment, the cross-section analysis was conducted on the brake material samples under 0 °C and −30 °C, respectively, see Figure 19, Figure 20, Figure 21 and Figure 22. Under intermittent braking conditions at 0 °C (Figure 19), large abscission pits appeared in the specimen portion of the brake pad material due to particle abscission, and graphite fracture and cracking of the brake pad material could be clearly observed. At −30 °C (Figure 20), the number and size of the abscission pit observed in the sample sections of the brake pad material were significantly reduced, and no subsurface cracks were found in the graphite surrounding the abscission pit; however, most of the detached particles were located between the different microstructures. Under the intermittent braking conditions at 0 °C, shown in Figure 21, a large abscission pit was observed on the surface of the brake disc material. At the ambient temperature of −30 °C, in Figure 22, the samples had relatively flat profiles. The results show that during the wear test, the surface material of the disc material is severely removed and worn at 0 °C. However, no plastic deformation was observed in the sample profiles at 0 °C and −30 °C.

## 4. Discussion

The friction and wear properties of intermittent braking of a high-speed train at low temperatures were studied from four aspects: friction coefficient, wear rate, surface morphology damage, and subsurface damage. As can be seen from Section 3.1, the instantaneous friction coefficient fluctuates greatly overall throughout the intermittent braking test. However, the average friction coefficient does not differ much, most likely because the single contact time of the brake disc/pad friction pair is short during the intermittent braking process. During the 10 min of equipment idling, the surface temperature of the friction pair gets lowered, prolonging the formation of the adhesion so that, once again, the sliding friction is equivalent to rubbing on a new flat surface with adhesion. It is particularly noteworthy that under the conditions of 0 °C and −10 °C, at about 7000 s, the instantaneous friction coefficient at both temperatures has the opposite law. This may be because, at 0 °C, the brake material appears as particles. Because the friction time is short, the particles are not broken, which leads to adhesion on the material surface, so the particles play a cutting role, which makes the instantaneous friction coefficient rise sharply. It is worth noting that a very high value of the coefficient of friction (0.85) occurs at approximately 8000 s at 0 °C. This is probably due to the presence of a larger particle at this point, which intensifies the cutting effect and causes a sudden increase in the coefficient of friction, and after a short period of friction, the particle breaks up into fine particles. It can be seen from Section 3.2 that the wear rate of the brake disc/pad material reaches the maximum at 0 °C, and the conclusion that the instantaneous friction coefficient reaches the maximum at 0 °C was also verified. It can be clearly seen from the figure that the brake disc is significantly more affected by the change in ambient temperature than the brake pad. With the decrease in temperature, the wear rate of brake materials generally shows a trend of increasing first and then decreasing. This may be due to the increase in brittleness of brake materials, the decrease in toughness [24], and the increase in the number of abscission pits and scratches during intermittent braking, leading to the increase in wear rate. However, with the continuous reduction in temperature, the speed of adhesion formation on the surface of the brake material accelerates to reach equilibrium, thus reducing the wear rate. The surface damage of the brake disc/pad specimen was analyzed and understood in Section 3.3. The surface damage of the brake pad material only included abscission pits, irregular scratches, adhesions, furrows, etc. The surface damage of the brake disc mainly included furrows, scratches, and adhesions. At 0 °C, there were large abscission pits and scratches on the brake’s surface, which was the main reason for the increase in the instantaneous friction coefficient and wear rate. Adhesion was found in the other four temperature environments, so the friction coefficient and wear rate decreased compared to the 0 °C environment. It can be seen from the analysis in Section 3.4 that obvious particle breakage and cracks occurred at 0 °C. Compared to a −30 °C environment, the surface of the brake material in a 0 °C environment had serious wear, indicating that intermittent braking under a 0 °C environment has the greatest damage to the surface of brake material. At the same time, it can be concluded from the analysis of the surface topography that the overall damage of the brake pad material is higher than that of the brake disc material, and the brake material is more damaged and more serious when braking in a low-temperature environment. Especially at 0 °C~−10 °C, the damage is most serious, which may be due to the falling particles, which may be separated from the surface in the future and adhere to the disc surface to form a serious chip effect. It shows that in the low-temperature environment, the brake material is more likely to produce a large area of furrow because of the chip.

## 5. Conclusions

Main conclusions:

1. The instantaneous friction coefficient of intermittent braking fluctuates greatly at different temperatures and reaches a maximum of 0.85 at 0 °C.
2. Under different temperature conditions, the wear rate of the brake disc/pad material for intermittent braking shows a trend of increasing first and then decreasing and reaches the maximum at 0 °C. The brake disc is more affected by the change in ambient temperature than the brake pad.
3. The brake disc material has three types of damage, including furrow, scratch, and adhesion. The brake pad material has four types of damage, including abscission pit, furrow, scratch, and adhesion. With the decrease in temperature, the adhesion damage of brake pad/disc material increases.
4. At 0 °C, the brake pad material has crack damage.

To summarize, the brake material has good friction and wear performance when operating at 20 °C. With the decrease in temperature, the surface wear form has changed significantly, leading to a change in wear rate and wear coefficient. Especially in the 0 °C environments, crack damage was found, indicating that the brake materials have poor friction and wear performance during the 0 ~−10 °C period. Therefore, it is recommended that high-speed trains avoid intermittent braking during the 0 ~−10 °C period as far as possible. For high-speed trains that operate in cold temperatures for long periods of time, there should be more frequent service intervals, and the number of times the brake discs are serviced should be greater than the number of times the brake pads are serviced.

## Figures and Tables

**Table 1:** Chemical composition of braking materials (wt%).

Element	C	Mn	Cr	Si	S	Cu	O	Fe
Brake pad material	6.75	-	3.47	-	1.13	38.85	20.31	Bal
Brake disc material	0.42	1.02	0.70	0.54	-	-	-	Bal

**Table 2:** Experimental parameter settings.

Brake form	Brake Time/min	Brake Pressure/MPa	Initial Braking Speed/m/s	Ambient Temperature/°C
Intermittent braking	120 (Interval of 10 min)	1.0	0.5	20, 0, −10, −20, −30
